# The Role of [18F]FDG PET-Based Radiomics and Machine Learning for the Evaluation of Cardiac Sarcoidosis: A Narrative Literature Review

**DOI:** 10.3390/medicina61091526

**Published:** 2025-08-25

**Authors:** Francesco Dondi, Pietro Bellini, Roberto Gatta, Luca Camoni, Roberto Rinaldi, Gianluca Viganò, Michela Cossandi, Elisa Brangi, Enrico Vizzardi, Francesco Bertagna

**Affiliations:** 1 Nuclear Medicine, Università degli Studi di Brescia and ASST Spedali Civili di Brescia, 25123 Brescia, Italy; 2 Nuclear Medicine, ASST Spedali Civili di Brescia, 25123 Brescia, Italy; pietro.bellini@asst-spedalicivili.it (P.B.); roberto.rinaldi@asst-spedalicivili.it (R.R.); michela.cossandi@asst-spedalicivili.it (M.C.); 3 Dipartimento di Scienze Cliniche e Sperimentali, Università degli Studi di Brescia, 25123 Brescia, Italy; roberto.gatta@unibs.it; 4 Clinical Engineering, ASST Spedali Civili di Brescia, 25123 Brescia, Italy; gianluca.vigano@asst-spedalicivili.it; 5 Cardiology Unit, ASST Spedali Civili di Brescia and Università degli Studi di Brescia, 25123 Brescia, Italy; elisa.brangi@unibs.it (E.B.); enrico.vizzardi@unibs.it (E.V.)

**Keywords:** cardiac sarcoidosis, PET, PET/CT, positron emission tomography, [18F]FDG, [18F]fluorodeoxyglucose, radiomics, machine learning, texture analysis, PET/MR

## Abstract

*Background/Objectives*: Cardiac sarcoidosis (CS) is an inflammatory cardiomyopathy with a strong clinical impact on patients affected by the disease and a challenging diagnosis. *Methods*: This comprehensive narrative review evaluates the role of [18F]fluorodesoxyglucose ([18F]FDG) positron emission tomography (PET)-based radiomics and machine learning (ML) analyses in the assessment of CS. *Results*: The value of [18F]FDG PET-based radiomics and ML has been investigated for the clinical settings of diagnosis and prognosis of patients affected by CS. Even though different radiomics features and ML models have proved their clinical role in these settings in different cohorts, the clear superiority and added value of one of them across different studies has not been demonstrated. In particular, textural analysis and ML showed high diagnostic value for the diagnosis of CS in some papers, but had controversial results in other works, and may potentially provide prognostic information and predict adverse clinical events. When comparing these analyses with the classic semiquantitative evaluation, a conclusion about which method best suits the final objective cannot be drawn with the available references. Different methodological issues are present when comparing different papers, such as image segmentation and feature extraction differences that are more evident. Furthermore, the intrinsic limitations of radiomics analysis and ML need to be overcome with future research developed in multicentric settings with protocol harmonization. *Conclusions*: [18F]FDG PET-based radiomics and ML show preliminary promising results for CS evaluation, but remain investigational tools since the current evidence is insufficient for clinical adoption due to methodological heterogeneity, small sample sizes, and lack of standardization.

## 1. Introduction

Sarcoidosis is a rare systemic pathology of unknown origin characterized by the presence of non-necrotizing granulomatous inflammation and fibrosis in tissues that can involve multiple organs and mainly affects lungs and lymph nodes [1,2,3,4,5,6,7,8]. Other possible localizations of the disease are the eyes, skin, and nervous system, and, more rarely, the heart [7,8,9,10].

Cardiac sarcoidosis (CS) is an inflammatory cardiomyopathy that appears when the disease involves the heart, and this pathological condition is characterized by serious consequences and an increase in the mortality rate [1,11,12,13,14,15]. In particular, the inflammation of the myocardium related to CS can be the cause of palpitations, presyncope, syncope, chest pain and, in advanced stages, heart failure. Interestingly, in 15% of the patients, sudden cardiac death is the first presentation of the disease. The cause of these conditions must be searched in the presence of re-entry phenomenon in areas of myocardial inflammation or granulomatous fibrotic tissue [16,17]. In fact, even though only 5% of patients with CS demonstrate symptoms and despite the low incidence of clinical manifestations, it can be considered as a potentially fatal disease because of associated ventricular arrhythmias and conduction system alterations [18]. A delayed diagnosis of CS is related to worse outcomes and its early detection is therefore desirable for precise and effective management even in subclinical individuals [1,5,7,14,15,18]. In addition, an accurate diagnosis is mandatory to target immunosuppressive medications, which can reduce myocardial inflammation and decrease the incidence of adverse events [14,15,19,20]. However, diagnosis of CS can be challenging since it relies on a combination of both clinical and imaging criteria [14,15,19,21,22]. The precise diagnosis can be established with an endomyocardial biopsy if non-caseating granulomas are identified. Nevertheless, an invasive approach is characterized by a high degree of risks and does not yield a significant improvement in sensitivity because myocardial involvement is patchy [18,23]. Beyond biopsy limitations, CS diagnosis remains challenging due to non-specific symptoms (e.g., arrhythmias, heart failure) that overlap with other cardiomyopathies, and the aforementioned patchy nature of myocardial involvement.

[18F]fluorodesoxyglucose ([18F]FDG) positron emission tomography/computed tomography (PET/CT) is a diagnostic tool that has proved its ability for the evaluation of different inflammatory conditions [24,25,26,27]. This imaging technique leverages the increased glucose metabolism of inflammatory cells, thus allowing for the detection of cellular inflammation. Current guidelines endorse 18F-FDG PET/CT for CS diagnosis through standardized visual and semiquantitative criteria (e.g., focal/diffuse uptake patterns, standardized uptake value [SUV] metrics). However, interobserver variability in visual assessment persists, creating a clinical need for more objective, quantitative approaches [1,28,29,30,31]. CS is visualized by the presence of intense cardiac uptake in the regions involved by the disease and it has been proposed that semiquantitative [18F]FDG PET parameters offer more objective and reproducible metrics for diagnosing and monitoring the disease [1]. Radiomics, in particular the so-called Hand-Crafted-Radiomics (HCR), refers to the extraction of specific quantitative features from images that can quantify the inhomogeneity between adjacent pixels or voxels and gray-level histogram or morphological features [32]. Similarly, machine learning (ML), as a subset of Artificial Intelligence (AI), focuses on the development of algorithms that enable computers to autonomously train linear and largely non-linear predictive models through a fully data-driven approach [33,34,35,36,37]. The added value of radiomics and ML in the assessment of different medical conditions is starting to emerge since different studies in this field of research have been produced [33,38,39,40]. Deep Learning Radiomics (DLR) represents an evolution of HCR, where features are not extracted using predefined formulas, but rather are learned directly from the specific images under analysis, typically through multilayer neural networks. Pros and cons of HCR and DLR are still under evaluation [41,42,43].

**Figure 1:**
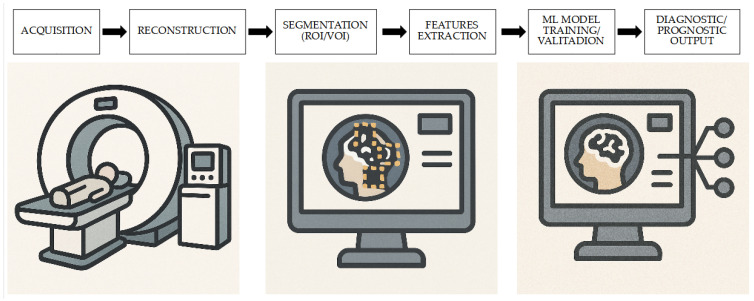
Radiomics workflow for CS: from PET acquisition to clinical decision. Starting from the acquisition of PET images and their reconstruction, the subsequent processing with segmentation and radiomics feature extraction is used to validate and train ML models with a possible prognostic or diagnostic value.

**Figure 2:**
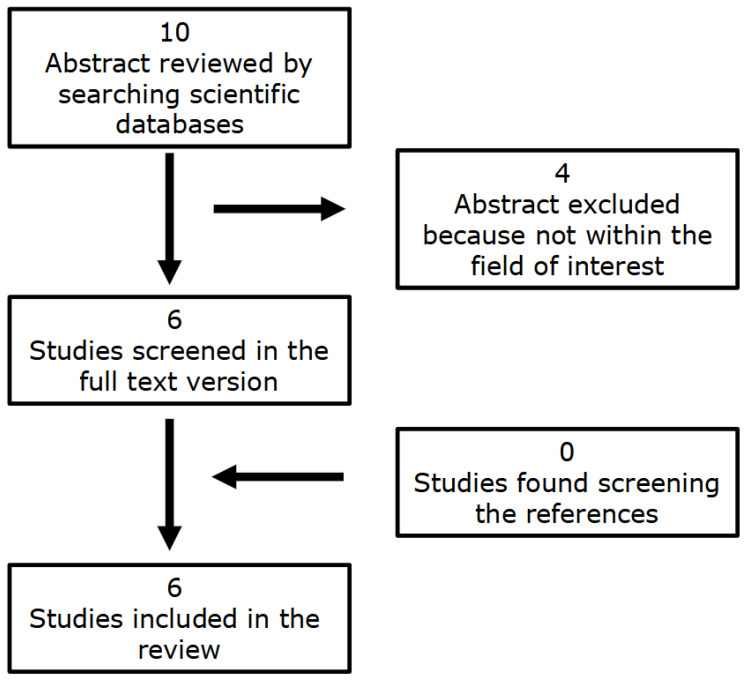
Flowchart of the research flow used for the review and to obtain eligible studies on the role of [18F]FDG-based radiomics and ML for the assessment of CS.

The aim of this comprehensive narrative review, therefore, is to evaluate the role of [18F]FDG PET-based radiomics and ML in the assessment of CS, summarizing the available literature in this field of research that is constantly gaining growing interest and which has recently experienced the publication of different papers (Figure 1 and Figure 2).

## 2. The Role of [18F]FDG PET-Based Radiomics and ML for the Evaluation of CS

Different studies assessed the role of texture analysis and ML derived from [18F]FDG PET imaging for the evaluation of CS in different settings and, interestingly, they were all published in recent years [44,45,46,47,48,49]. In particular, these papers focused on two different clinical settings: the diagnosis of CS and the prognostic evaluation of patients affected by the disease. The main characteristics of these studies are presented in Table 1, Table 2, Table 3 and Table 4.

### 2.1. Diagnosis

Manabe et al. [44] evaluated the ability of radiomics analyses (HCR) to help in the diagnosis of CS. Starting from inter-operator reproducibility, the authors revealed that classic semiquantitative parameters such as maximum SUV (SUVmax), cardiac metabolic volume (CMV), and cardiac metabolic activity (CMA) had high reproducibility (*p* < 0.0001 for all of them). Regarding radiomics features, 28/36 of them demonstrated significantly high reproducibility with intraclass correlation coefficients (ICC) over 0.80. The study was performed by using two different scanners; therefore, a comparison was proposed: no significant differences were reported for SUVmax, CMV, and CMA values and 17/36 textural features had ICC values over 0.80. The ability of semiquantitative PET parameters to differentiate between CS and non-CS patients was evaluated, and CMV and CMA, but not SUVmax, were significantly different between the two groups (*p* = 0.0002 and *p* = 0.0001, respectively). Almost all the radiomics features could distinguish CS from non-CS and the area under the curve (AUC) values of 16/36 of them showed remarkable performance (AUC > 0.80). After hierarchical clustering, eight features were selected to be included in the multivariate logistic regression analysis, concluding that long-run emphasis (LRE) and short-run low gray-level emphasis (SRLGE) were significant independent factors that distinguished between CS and non-CS (*p* = 0.0004 and *p* = 0.016, respectively). LRE demonstrated the highest diagnostic ability (AUC 0.91) and a high inter-operator reproducibility (ICC 0.98). When the cohort was divided into CMV > 10 milliliters (mL) and CMV ≤ 10 mL groups, LRE was still significantly different between CS and non-CS subjects in both of them.

Togo et al. [45] investigated the information content of polar maps through a DLR approach. They built a deep convolutional neural network (DCNN)-based method to classify CS and non-CS patients and compared it with SUVmax-based and coefficient of variation (CoV)-based classifications, revealing that their signature method had the best sensitivity, specificity, and harmonic mean (0.839, 0.870, and 0.854, respectively), in particular when the ReliefF algorithm was applied to select features. More precisely, 26/33 (78.8%) CS patients and 47/52 (90.0%) of the non-CS subjects were correctly classified. When considering only the top 10-dimensional features, the sensitivity, specificity, and harmonic mean were 0.741, 0.666, and 0.722, respectively. These performances were roughly equivalent to the performance of the CoV-based classification method.

Mushari et al. [46] assessed the ability of radiomics analysis on handcrafted features based on [18F]FDG PET/cardiac magnetic resonance (CMR) imaging to improve the diagnostic accuracy of CS. The authors investigated and compared two different segmentation methods for the extraction of radiomics features and conventional metrics: for method A, a region of interest (ROI) was drawn in the hot region of the myocardium with an SUV higher than 2.5 and, in the case of multiple lesions, the largest and most active one was selected. In controls, the ROI was created in the normal myocardium. Target-to-background ratio (TBR) with the blood-pool of the right atrium was calculated. Unlike method A, method B was independent from intensity and pattern since an ROI was drawn in the entire left ventricle. For segmentation A, SUVmin, gray-level co-occurrence matrix (GLCM)_inverse difference normalized, and gray-level size zone matrix (GLSZM)_large area high gray-level emphasis had the highest AUC (1.00 for all of them), while for segmentation B, the best performances were obtained by SUV 90 percentile, SUVmax, and TBRmax (0.90, 0.90, and 0.96 respectively) and, in general, the textural features had similar or lower AUCs. Interestingly, 40/75 textural features for method A and 61/75 features for method B were significantly different between CS and non-CS subjects (*p* < 0.00061). After applying a logistic regression classifier to reduce redundancy, only 22 radiomics features for segmentation A and 35 for segmentation B fulfilled the following criteria: *p* < 0.00061, AUC > 0.5, and accuracy > 0.7. Five principal component features were retained to build a ML classifier: for segmentation A, all classifiers showed high performance in terms of AUC (95% confidence interval [CI] 0.88–1.00) and accuracy (95% CI 0.87–1.00), with values > 0.9. K-neighbors and neural network classifiers showed the highest AUC and greatest accuracy, with values equal to 1.00. For segmentation B, four classifiers with AUCs and accuracies > 0.8 were built.

Lastly, Kote et al. [47] evaluated the role of textural features extracted from [18F]FDG PET imaging to diagnose CS. The authors reported that neither SUVmax nor SUVmean were significantly different between the CS and non-CS groups. Focusing on first-order parameters, only discretized_ histo_entropy was significantly different between the two groups, with an AUC of 0.79 at a cutoff of 0.1092. Among the second-order parameters, four from the gray-level run length matrix (GLRLM) and four from the gray-level zone length matrix (GLZLM) were significantly different between the CS and non-CS subjects: GLRLM_LRE, GLRLM_LGRE, GLRLM_SRLGE, GLRLM_LRLGE, GLZLM_LZE, GLZLM_LGZE, GLZLM_SZLGE, and GLZLM_LZLGE (AUC > 0.7 for all of them). In the case of higher-order parameters, GLCM_homogeneity, GLCM_energy, and neighborhood gray-level difference matrix (NGLDM)_coarseness were significant in differentiating patients with CS and normal controls (AUC > 0.7 for all of them). After testing five different ML methods, the gradient boosting classifier gave the best performances on these parameters with 85.71% accuracy and an F1 score of 0.86 on both classes.

Notably, while conventional SUV metrics (SUVmax, SUVmean) showed limited diagnostic value in some studies [44,47], CMV and CMA demonstrated significant differences between the CS and non-CS groups [44]. This fact may suggest that volumetric parameters may better capture disease burden than intensity-based metrics. Among radiomics features, LRE and SRLGE emerged as robust diagnostic indicators across studies [44,47], with AUCs > 0.90 in independent cohorts. Contradictions in feature performance (e.g., GLCM_homogeneity vs. GLCM_inverse difference normalized) likely reflect methodological variations rather than true biological discordance, underscoring the need for standardized feature extraction protocols.

### 2.2. Prognosis

Manabe et al. [48] evaluated 62 patients affected by CS by extracting 36 textural features from [18F]FDG-derived polar maps. No significant differences were reported between SUVmax, CMV, or MCA between patients with different prognoses on the basis of major adverse cardiac events (MACE). In contrast, high gray-level run emphasis (HGRE) was significantly higher in patients with MACE than in patients without MACE (*p* 0.004). Univariate analysis revealed that the New York Heart Association (NYHA) functional classification, left ventricular ejection fraction, and HGRE were significantly associated with MACE. At multivariate Cox proportional hazards models, HGRE was significantly associated with MACE after adjusting for left ventricular ejection fraction (LVEF) or NYHA classification (*p* < 0.05 for both).

More recently, Nakajo et al. [49] evaluated the ability of ML, radiomics features, and the visibility of the right ventricle (RV) at [18F]FDG PET/CT to predict the clinical events and the response to treatment of 47 patients with CS. The complication rate of adverse clinical events (ACE) was significantly higher in patients with RV uptake (85.7% [6/7] vs. 27.5% [11/40] for negative RV, *p* = 0.006). When dividing the patients into training and testing cohorts, no significant differences were reported in terms of RV uptake and ACEs between the two groups (*p* > 0.05 in all the cases). The best radiomics features to predict ACEs were surface area, GLRLM_RLNU, neighborhood gray tone difference matrix (NGTDM)_coarseness, and sphericity. Patients who experienced ACEs had significantly higher surface area (*p* < 0.001) and GLRLM_RLNU (*p* < 0.001) and lower NGTDM_coarseness (*p* = 0.002) and sphericity (*p* = 0.010) than those without ACEs. In the training cohort, all the different ML algorithms that were built by the authors achieved AUC > 0.80 (0.841–0.944) for the prediction of ACEs. Five of seven algorithms (decision tree, random forest [RF], neural network, logistic regression [LR], and support vector machine [SVM]) achieved F1 scores (range: 0.812–0.875), precision (range: 0.817–0.886), recall (range: 0.813–0.875), and accuracy (range: 0.813–0.875) of >0.80. In the testing cohort, only RF and neural network had an AUC > 0.80. The performances of RF (AUCs 0.935 and 0.889 in the training and testing cohorts, respectively) and neural network (AUCs 0.944 and 0.889 in the training and testing cohorts, respectively) in the testing cohort were similar to that of the training cohort. The performances of the remaining five ML algorithms were poorer in the testing cohort (AUCs 0.667–0.778) than in the training cohort. Sensitivity, specificity, positive predictive value (PPV), negative predictive value (NPV), accuracy, and AUC were not significantly different among the seven ML algorithms (*p* > 0.05 for all of them). Despite that, RF had the highest diagnostic index (AUC 0.889, F1 score 0.882, precision 0.905, recall 0.899, sensitivity 66.7%, specificity 100%, PPV 100%, NPV 85.7%, and accuracy 88.9%). GLRLM_RLNU was the most important feature with a higher contribution in the modeling process.

Prognostically, HGRE and GLRLM_RLNU were consistently associated with adverse events, independent of functional parameters like LVEF [48,49]. These features may reflect inflammation heterogeneity linked to fibrosis progression. While multiple ML algorithms (random forests, neural networks) achieved high AUCs (>0.88), no single model demonstrated clear superiority across cohorts. Algorithm-agnostic approaches focusing on biological relevance rather than model architecture are recommended for future studies.

## 3. Discussion

The aim of this review was to evaluate the possible added value of [18F]FDG PET-based radiomics and ML for the evaluation of CS by summarizing the available literature on this topic. Different papers have explored this subject, suggesting a potential role for these approaches in the diagnosis and the prognostic assessment of patients affected by this pathological condition [44,45,46,47,48,49]. In the papers by Manabe et al. [44] and Togo et al. [45], interesting and useful visual representations of the processes and the possible value of radiomics and ML in the assessment of CS can be found. Different radiomics features have been proposed as useful to evaluate CS; however, in general, different papers reported different textural parameters as the ones with best performances. However, in two papers that focused on the diagnostic ability of [18F]FDG imaging, there was a limited coincidence of the radiomics features group with best performances: GLCM is a second order class which contains spatial information about the relationship of pixel pairs with similar or specific intensity within an image [50]. Despite this small coincidence, Kote at al. [47] reported GLCM_homogeneity and GLCM_Energy as the features with best performances, while Mushari et al. [46] reported GLCM_inverse difference normalized as the feature with the best AUC. Furthermore, GLRLM describes the information about the number of runs of pixels with the same intensity in a specific direction [50] and also this group of features has been cited in two different papers: Kote et al. [47] reported GLRLM_LRE, GLRLM_LGRE, GLRLM_SRLGE, and GLRLM_LRLGE as features with high performances for the diagnosis of CS, while Nakajo et al. [49] underlined GLRLM_RLNU as the most important feature for a modeling process of prognostic value. Although the features are nominally different, it is important to note that the authors reported only those that yielded the best results in their analyses. The different results should therefore not necessarily be regarded as contradictory. It is reasonable to assume (though this would require further investigation) that these features are significantly correlated with one another, and that the nominal differences are mostly due to slight variations in the datasets.

Conventional semiquantitative PET parameters have been suggested in the past as useful to assess CS [29,30,51,52]. In particular, it has been reported that [18F]FDG PET imaging has moderate sensitivity and specificity for the diagnosis of the disease, with pooled values of 0.84 and 0.83, respectively [53]. Additionally, when comparing CMR and [18F]FDG PET, they had sensitivities of 92% and 81% and specificities of 72% and 82%, respectively. Interestingly, the sensitivity of [18F]FDG PET was highest when using quantitative analysis if compared to qualitative evaluation (93% vs. 76%) [54]. Some papers included in the present review attempted to make a comparison between the diagnostic performances of conventional semiquantitative parameters and radiomics features; however, in some analyses these features performed better than conventional SUV parameters, while in other papers this difference was not reported [44,46,47,48]. Therefore, a final conclusion on which analysis best suits the final objective cannot be drawn with the currently available references.

One of the main points that is mandatory to underline when performing a general analysis of the papers included in the review, in the attempt to compare their findings, is the fact that different segmentation protocols and methods to extract the radiomics features were used among the studies. For instance, studies used divergent approaches: whole-left ventricle volume of interest [45,46,47] vs. lesion-specific ROIs [44,47], with thresholds ranging from SUV > 2.5 to 40% of the SUVmax. Focusing on segmentation, this procedure was in some cases performed by comprehending all the left ventricle, while in other papers only “hot regions” were considered and included in ROIs. Furthermore, in some cases only the region with the highest uptake was considered, while in others all the regions with increased tracer uptake were included. In this setting, it is known that the cardiac involvement by sarcoidosis can be focal, multifocal, or diffuse [51,53,55]; therefore, considering different methods of segmentation can be a major issue for the extraction of radiomics features and for the validation of the findings reported in a single paper. We recommend adopting SNMMI/ASNC consensus standards for myocardial segmentation and SUV thresholding to improve reproducibility [29]. Additionally, in different cases, textural features were extracted by generating a polar map from the tracer distribution inside the myocardium, while in other papers the features were directly extracted from [18F]FDG images. Interestingly, a single paper described the use of PET/CMR instead of PET/CT, and this fact may introduce spatial resolution differences, potentially altering texture feature distributions, a variable requiring systematic evaluation [46]. Again, all together, these are important issues that can impact the generalizability of our findings, thus limiting the values of the data reported in the present review.

Another interesting point is that the regions of CS characterized by intense uptake of [18F]FDG were in some papers identified by a visual analysis while in other cases an SUV-based threshold was used. As previously mentioned, conventional SUV parameters have demonstrated their value in the assessment of CS, and the joint Society of Nuclear Medicine and Molecular Imaging (SNMMI)/American Society of Nuclear Cardiology (ASNC) consensus expert document recommends their use to quantify the intensity and the amount of myocardial inflammation related to the presence of CS, even in the monitoring of response to therapy [29,30,51]. In this scenario, it has been reported that even though a specific and rigorous head-to-head comparison between different SUV metrics has been proposed, the available evidence suggests that they perform better than the qualitative visual assessment of [18F]FDG images to evaluate the treatment response [30,51,56,57,58].

ML is based on the learning aspects of artificial intelligence by focusing on its ability to learn from and to represent a set of data [34,35,59,60]. In general, no specific ML algorithm has been confirmed in different studies as the one with the best performances. Furthermore, all the radiomics analyses proposed in the papers are affected by different issues, resulting in a low level of the evidence reported. As a matter of fact, small cohorts were used and the balance between positive and negative cases was often far from a 1:1 ratio. These facts impact the reproducibility and the generalizability of the findings reported in the present review, reducing the possible clinical applications of radiomics and ML in the evaluation of CS.

Before clinical implementation, three barriers must be addressed to endorse the use of radiomics and ML: first of all, external validation in multicentric trials using harmonized PET protocols is necessary. Development of open-source, vendor-agnostic radiomics pipelines to overcome computational resource limitations and integration of radiomics with clinical variables (e.g., NYHA class) and serological biomarkers are other point that must be considered. Crucially, radiomics should complement and not replace established semiquantitative PET parameters until prospective validation confirms incremental value.

Several intrinsic problems that need to be overcome in the future are present in radiomics and ML technologies. Reproducibility and repeatability are major issues of radiomics feature extraction and subsequent analysis; these are well-known topics in current literature and future efforts in the direction to resolve them need to be performed. Focusing on the specific setting of PET imaging, it has been reported that different scanners, tumor segmentation, partial volume effect, reconstruction protocols, and uptake time can influence the extraction of textural features and subsequent ML analyses [35,61,62,63,64,65,66,67,68]. In particular, the use of different scanners with different reconstruction protocols of PET images has been related to the different sensitivities of radiomics features. Large variations in their quantification can be present when using different reconstruction parameters and algorithms, even though some classes of textural features exhibited a higher robustness among different protocols [58,69,70,71]. For example, it has been underlined that the full width at half maximum of the gaussian filter and the iteration number have a similar impact on these features, while grid size has a larger influence [58,59,60,61,62,63,64,65,66,67,68,69,70,71]. Additionally, it has been also reported that there is a high instability exhibited by 19%, 33%, and 36% of radiomics features with a coefficient of variation of more than 20% for reconstruction, segmentation, and discretization when focusing of [18F]FDG PET/CT in the setting of lung cancer assessment [72].

This review is affected by different limitations that are directly derived by the studies included. First of all, all of them are retrospective analyses based on single-center settings, an issue that can therefore influence the generalizability of their findings, which need to be confirmed in prospective multicentric protocols. Furthermore, the use of different methods for the segmentation and the extraction of radiomics features can impact their general value for the assessment of CS. As mentioned, radiomics and ML technologies are characterized by intrinsic issues. Moreover, an external validation was not included in the papers cited in the review, a point that is mandatory to strengthen the value of the results obtained in monocentric retrospective settings [35,73,74,75]. Lastly, as previously underlined, one of the major issues that is present in most of the papers analyzed is the fact that they were performed on limited cohorts and samples, which is known to be a big issue for a clear evaluation of the potential and the value of radiomics analyses in CS. In this scenario, the data summarized in this review are heterogeneous and seem not to endorse a clear indication of the added value of [18F]FDG PET-based radiomics and ML for the evaluation of CS. Their applications in routine clinical settings are hard to implement since the aforementioned limitations may impact this process. Future research is therefore needed to clearly evaluate the added value of these analyses in the specific setting of the review and to eventually implement these technologies in daily clinical life.

## 4. Conclusions

Our review underlines how [18F]FDG PET-based radiomics and ML show preliminary promise for CS evaluation but currently remain investigational tools. Current evidence is insufficient for clinical adoption due to methodological heterogeneity, small sample sizes, and lack of standardization. Future research must prioritize different points, such as multicentric settings with protocol harmonization, the development of disease-specific radiomics signatures validated against histopathological endpoints, and cost-effectiveness analyses comparing radiomics-enhanced workflows against standard care. Until these steps are achieved, conventional PET parameters remain the evidence-based quantitative standard. The potential clinical implications of adopting radiomics and ML in the assessment of CS lie in the support that these technologies can offer in improving disease diagnosis, achieving a more precise characterization through non-invasive methods, and potentially enhancing the prediction of patient prognosis.

## Figures and Tables

**Table 1:** Characteristics of the studies considered for the review.

First Author	N. Ref.	Year	Country	Study Design	N. Pts.	CS Pts. (%)	Setting
Manabe O	[44]	2018	Japan	Retrospective	89	37 (42)	Diagnosis
Togo R	[45]	2019	Japan	Retrospective	85	33 (39)	Diagnosis
Manabe O	[48]	2020	Japan	Retrospective	62	62 (100)	Prognosis
Mushari NA	[46]	2022	UK, USA, Netherlands	Retrospective	69	40 (58)	Diagnosis
Kote R	[47]	2024	India	Retrospective	67	17 (25)	Diagnosis
Nakajo M	[49]	2024	Japan	Retrospective	47	47 (100)	Prognosis

N.: number; Pts: patients; Ref.: reference; USA: United States of America; UK: United Kingdom; CS: cardiac sarcoidosis.

**Table 2:** Characteristics of the papers included in the review.

First Author	N. Ref.	Device	Number of Scanners	Scanner Type	Reconstruction Protocol	Reported Activity (MBq)	Cardiac Uptake Suppression
Manabe O	[44]	PET/CT	1	Siemens Biograph 64 TruePoint TrueV	PSF	4.5/kg	LCD
Togo R	[45]	PET/CT	1	Siemens Biograph 64 TruePoint TrueV	PSF	4.5/kg	LCD
Manabe O	[48]	PET/CT	1	Siemens Biograph 64 TruePoint TrueV	PSF	4.5/kg	LCD, unfractionated heparin
Mushari NA	[46]	PET/CMR	1	Siemens BiographTM mMR	OSEM	5/kg	LCD
Kote R	[47]	PET/CT	1	GE Discovery MI-DR	ns	ns	LCD
Nakajo M	[49]	PET/CT	2	GE Discovery 600M, GE Discovery MI	OSEM, PSF	223 ± 30	LCD

PET: positron emission tomography; CT: computed tomography; MBq: megabecquerel; Ref: reference; kg: kilogram; CMR: cardiac magnetic resonance; ns: not specified; LCD: low-carbohydrate diet; PSF: point spread function; OSEM: ordered subset expectation maximization.

**Table 3:** Results and main findings of the studies considered for the review.

First Author	Ref.	Performance Validation Methods	ML Models	Class Balancing	Main Findings
Manabe O	[44]	Train/test	Logistic regression	42/58	Some textural features showed high diagnostic value for CS diagnosis.
Togo R	[45]	Cross-fold	Deep convolutional neural network	33/52	Radiomics features may be more effective than conventional semiquantitative features for CS diagnosis.
Manabe O	[48]	Train/test	Logistic regression	ns	[18F]FDG textural features may potentially provide prognostic information in CS subjects.
Mushari NA	[46]	Cross-fold	Random Forest, Logistic Regression, Support Vector Machine, Decision Tree, Gaussian Process Classifier, Stochastic Gradient Descent, Perceptron Classifier, Passive Aggressive Classifier, Neural Network Classifier and K-neighbors Classifier	ns	Radiomic analysis of PET data may not be a useful approach to detect CS. Conventional semiquantitative parameters show high performances.
Kote R	[47]	ROC	ns	ns	Textural analysis parameters could successfully differentiate CS from non-CS.
Nakajo M	[49]	Train/test	Decision tree, random forest, neural network, k-nearest neighbors, Naïve Bayes, logistic regression, and support vector machine	38/9	ML analyses using [18F]FDG PET-based radiomics features may be useful to predict adverse clinical events in CS subjects.

Ref.: reference; [18F]FDG: [18F]fluorodeoxyglucose; ns: not specified; ML: machine learning; CS: cardiac sarcoidosis; PET: positron emission tomography; ROC: receiver operating characteristics.

**Table 4:** Summaries of diagnostic workflow used in the different studies.

First Author	N. Ref.	Models
Manabe O	[44]	Selection of [18F]FDG avid areas in the left ventricle Extraction of SUVmax, CMV, CMA Generation of short-axis images and reconstruction of polar map with linear interpolation method Extraction of radiomics features from polar map
Togo R	[45]	Selection of [18F]FDG avid areas in the left ventricle Extraction of SUVmax Generation of short-axis images and reconstruction of polar map with linear interpolation method Extraction of features from the polar map and selection Evaluation of effectiveness of DCNN-based features with SVM classifier
Manabe O	[48]	Selection of [18F]FDG avid areas in the left ventricle Extraction of SUVmax, CMV, CMA Generation of short-axis images and reconstruction of polar map with linear interpolation method Extraction of radiomics features from polar map
Mushari NA	[46]	Segmentation A: ROI drawing in hot regions with SUV > 2.5 and evaluation of TBR; segmentation B: ROI drawing on the entire left ventricle Extraction of conventional and radiomics metrics Application of ML models
Kote R	[47]	Drawing of ROIs on the myocardium with 40% thresholding Extraction of conventional and textural features Passing of these features through a principle component analysis algorithm Testing of ML classifiers
Nakajo M	[49]	Generating VOI by placing ROI on a reference-fused axial image and applying a 40% threshold of SUVmax Extraction of radiomics features after harmonization for different scanners Application of ML models

Ref.: reference; [18F]FDG: [18F]fluorodeoxyglucose; SUVmax: maximum standardized uptake value; CMV: cardiac metabolic volume; CMA: cardiac metabolic activity; DCNN: deep convolutional neural network; SVM: support vector machine; ROI: region of interest; TBR: target-to-background ratio; ML: machine learning; VOI: volume of interest.

## Data Availability

Data supporting the reported results can be found using the public scientific databases.
